# Citicoline for Supporting Memory in Aging Humans

**DOI:** 10.14336/AD.2022.0913

**Published:** 2023-08-01

**Authors:** Maciej Świątkiewicz, Paweł Grieb

**Affiliations:** Department of Experimental Pharmacology, Mossakowski Medical Research Institute, Polish Academy of Sciences, Warsaw, Poland

**Keywords:** citicoline, humans, aging, cognitive ability

## Abstract

Citicoline is the generic name of CDP-choline, a natural metabolite presents in all living cells. Used in medicine as a drug since the 1980-s, citicoline was recently pronounced a food ingredient. When ingested, citicoline breaks down to cytidine and choline, which become incorporated into their respective normal metabolic pathways. Choline is a precursor of acetylcholine and phospholipids; these is a neurotransmitter pivotal for learning and memory and important constituents of neuronal membranes and myelin sheaths, respectively. Cytidine in humans is readily converted to uridine, which exerts a positive effect on synaptic function and supports the formation of synaptic membranes. Choline deficiency has been found to be correlated with memory dysfunction. Magnetic resonance spectroscopy studies showed that citicoline intake improves brain uptake of choline in older persons, suggestive of that it shall help in reversing early age-related cognitive changes. In randomized, placebo-controlled trials of cognitively normal middle-aged and elderly persons, positive effects of citicoline on memory efficacy were found. Similar effects of citicoline on memory indices were also found in patients suffering from mild cognitive impairment and some other neurological diseases. Altogether, the aforementioned data provide complex and unambiguous evidence supporting the claim that oral citicoline intake positively influences memory function in humans who encounter age-related memory impairment also in the absence of any detectable neurological or psychiatric disease.

## Introduction

Memory is the cognitive ability to maintain previously learned information so that it may be accessed and used at a later time. It is not a unitary construct but reflects a number of distinct cognitive processes (e.g., episodic memory, working memory, short-term memory, semantic memory, etc.). In the extended definition, memory is the capacity to store and retrieve information [1].

Although aging is characterized by the development of a broad spectrum of pathologies, traditionally, it has been viewed as a natural process and, consequently, not a disease. When aging is not complicated by neurological or psychiatric disease, cognitive functions are not impaired but become slowed. In particular, in middle-aged and elderly people the memory and its constituents are noticeably failing, which is considered physiological. Almost everybody will subjectively experience certain deterioration of memory with aging, manifested *inter alia* as prolongation of time required to memorize new information and to recall information previously remembered. A frequently encountered aspect of age-related failing of human memory is the impairment of episodic memory, i.e., the memory of everyday events that can be explicitly stated or conjured (for example, times, location geography, associated emotions, and other contextual information) [2]. The maintenance or reduced loss of one or more cognitive processes related to memory is considered a beneficial physiological effect [3].

Citicoline is the generic name of a substance identical to cytidine-5’-diphosphocholine (CDP-choline), a natural metabolite presents in minute amounts inside every living cell and playing a crucial role in the synthesis of cellular phospholipids [4]. CDP-choline from an exogenous source, known as citicoline, has been used in medicine since the 1970-s as a prescription drug displaying neuroprotective effects, indicated for various chronic and acute neurological diseases (e.g., Parkinson’s disease, stroke, brain and spinal cord injury, glaucoma). [5]. More recently, one of its variants (citicoline inner salt) was pronounced a food constituent in the major world markets (USA, European Union) [6]. Therefore, citicoline became freely available over the counter as a food supplement that is considered a memory enhancer [7]. The aim of the present review is to critically discuss the evidence for positive effects of this substance on memory in middle-aged and elderly persons who do not suffer from any neurological or psychiatric disease. In humans, citicoline following ingestion is well absorbed and rapidly broken down into choline and cytidine. This results in a marked increase of blood choline level, whereas blood cytidine is quickly converted to uridine. Next, choline and uridine enter cells of the human body and join their appropriate metabolic pathways. In an analogy to the term “a prodrug” used in pharmacy to depict a compound which is metabolized in the body to produce an active drug, citicoline shall be called “a pro-nutrient” that in the human body produces choline and uridine as “active nutrients”.

**Figure 1.**
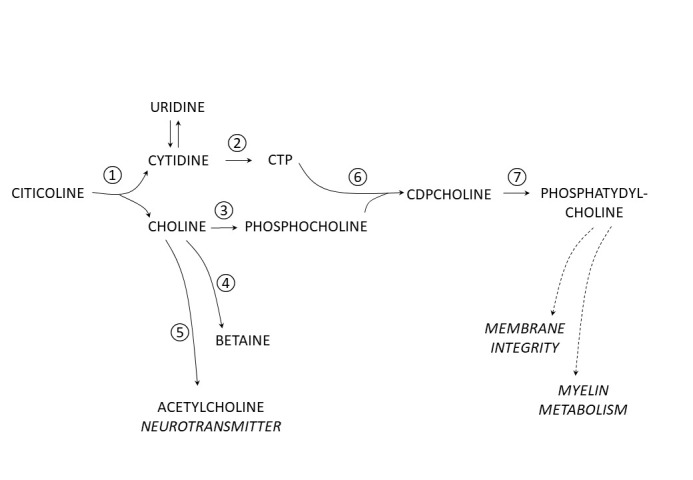
**Major metabolic pathways of citicoline following ingestion**. 1. Hydrolysis and dephosphorylation of citicoline occur in blood and yield choline and cytidine (in humans converted to uridine) that enter brain. 2. In the brain cytidine is activated by phosphorylation to cytidine-triphosphate (CTP). Choline, which concomitantly enters the brain, follows three major paths: 3. activation by phosphorylation to phosphocholine; 4. conversion to betaine (N,N,N-trimethylglycine); 5. conversion to acetylcholine (a neurotransmitter). 6. CTP and phosphocholine rejoin to yield CDP-choline, which 7. is used to synthesize phosphatidylcholine, utilized to support membrane integrity and myelin metabolism.

The metabolic fate of citicoline and its hydrolysis/ dephosphorylation products have been extensively studied in experimental animals (see [8], and the references cited). In the central nervous system, the three most prominent pathways (schematically shown on Fig. 1) include (a) the synthesis of the neurotransmitter acetylcholine, (b) the aforementioned “Kennedy pathway” that also utilizes cytidine and leads to the synthesis of phosphatidylcholine, and (3) oxidation of betaine, providing one-carbon unit for the conversion of homocysteine to methionine.

During decades of medical use, it became apparent that the application of citicoline is not linked to any significant adverse effects, and no contraindication to its use has ever been indicated. Its almost perfect tolerance observed in human studies has been corroborated by results of a subchronic toxicological study performed on rats in which a dose of 1 gram per kg body weight per day given orally for 3 months produced only slight signs of toxicity related most likely to excess of phosphate [9]. In various clinical studies, patients were receiving daily citicoline doses of up to 2 grams, given either parenterally or orally. Recommended maximal dosing of citicoline (inner salt) as a food supplement is 500 mg per day and as dietary foods for special medical purposes as 1,000 mg.

## Citicoline effects on human memory in elderly individuals

References related to the scientific data pertinent to the issue of citicoline and memory in humans were identified with Pubmed, Scopus, and Web of Science databases, using a composition of key words: “citicoline pharmacokinetics and human”, “citicoline and human memory”, “choline and human memory”, and “uridine and human memory”. When required, the references concerning the aforementioned combinations of keywords are supplemented by the appropriate references concerning pertinent general issues, such as “definitions of the human memory”, “healthy aging” compared to “age-related pathology”, etc.

Only human studies were taken into account for two main reasons: First, human memory and related cognitive functions are qualitatively different from the memory and cognitive functions of laboratory animals (mostly rodents). Consequently, methods used to investigate the efficacy of human memory and cognition are vastly different from those used for testing the efficacy of memory of laboratory mice and rats, therefore extrapolation of the data from preclinical experiments to humans would not be appropriate. Second, in animal studies, citicoline and/or its catabolites (choline and cytidine/uridine) were almost always tested in doses severalfold larger than those that could be taken by humans, making extrapolation of the animal data to humans doubtful. Results of animal experiments are cited only exceptionally.

There are four aspects of the scientific substantiation of the positive effect of oral citicoline intake on the maintenance, reduced loss, or improvement of memory in healthy middle-aged and elderly persons. The first aspect is related to choline insufficiency in age-dependent memory deterioration. Although choline is synthesized in the human body, its *de novo* synthesis is not adequate to meet the demand for this substance, making it an essential nutrient. The best-known effect of a choline-deficient diet, occurring concomitantly with diminished blood plasma choline and phosphatidylcholine concentrations, is the development of liver damage which is reversed by the reintroduction of choline supply. There is limited data concerning a similar “depletion-repletion” effect of choline on human memory and cognition. However, there is convincing evidence from large population studies that the availability of dietary choline is positively correlated with memory efficiency in adults and the elderly. The second aspect is related to the role of uridine as a precursor for phospholipids of neuronal cell membranes. This issue is relevant because in humans (unlike in rodents), cytidine released from citicoline after its oral intake is in circulating blood plasma readily and almost completely converted to uridine. The third aspect is related to the studies of choline uptake by the human brain following the intake of choline and citicoline and its metabolic disposal. Intensification of the brain choline uptake has been shown of particular importance in middle-aged and older human individuals. The fourth aspect focuses on interventional trials assessing oral intake of citicoline on memory in middle-aged or elderly persons considered healthy.

The references pertaining to the scientific substantiation of the effect of citicoline on memory functions in healthy middle-aged and older persons are supplemented by the selected references concerning the effects of citicoline on the memory of patients suffering from certain chronic neurological diseases. These data are presented as a part of the totality of the evidence for support of the claim concerning citicoline and human memory because citicoline effects on memory in these patients and in cognitively healthy elderly persons suffering from subjective memory impairment seem to be similar.

## Choline insufficiency in age-dependent memory deterioration of healthy individuals

As previously mentioned, citicoline following ingestion is well absorbed and rapidly broken down into choline and cytidine, which in humans is quickly and almost completely metabolized to uridine. Food-borne choline and other choline-containing food ingredients (carnitine, betaine, etc.) are partially metabolized by intestine bacteria to a gaseous metabolite trimenthylamnie (TMA), which is further transformed in the liver to its oxide (TMAO). The latter substance is suspected to be atherogenic, and recently it has also been implicated in the development of Mild Cognitive Impairment and Alzheimer’s Disease [10,11]. Choline derivatives from ingested food may have different accessibility for the gut microbes due to their different moieties and intestinal absorption rates. It has been suggested that citicoline is a potentially safer form of choline because it may resist hydrolysis inside the intestinal lumen, possibly due to its pyrophosphate group [6].

In humans as well as in animal choline is a substrate for the synthesis of acetylcholine (ACh), the main neurotransmitter on which important neuronal circuits involved in memory depend. ACh is widely distributed in the nervous system and has been implicated to play a critical role in many aspects of brain functioning, including modulating cognitive performance, learning and memory processes. The basal forebrain cholinergic complex (comprising medial septum, horizontal and vertical diagonal band of Broca, and nucleus basalis of Meynert) provides the major cholinergic projections to the cerebral cortex and hippocampus. The synthesis of the neurotransmitter ACh in cholinergic neurons depends on the availability of its precursor choline, most of which is derived from the circulation and enters cholinergic neurons via a process catalyzed by a specific transporter (reviewed in [12]).

Animal studies indicate that citicoline may increase choline availability to cholinergic neurons not only directly by increasing blood plasma choline level, but also indirectly by modulating the expression of proteins relevant to ACh metabolism. In experiments on rats one week of daily intraperitoneal (i.p.) injections of citicoline (or the other choline containing compound, choline alfosclerate) resulted in enhanced expression of choline transporter (ChT) responsible for delivering choline to ACh-synthesizing neurons and vesicular acetylcholine transporter (VAChT) responsible for loading ACh into presynaptic vesicles [13]. The other mechanism by which citicoline could support memory could relate to enhanced sirtuin-1 (SIRT1) expression, which was observed in the rat brain one day after a single i.p. injection of citicoline [14]. In mice, normal cognitive function and synaptic plasticity were impaired by knocking out SIRT1 gene [15]. However, these effects may indicate advanced pathological rather than physiological brain aging. The level of SIRT1 protein in the human brain cortex investigated post-mortem appeared to decrease in Alzheimer’s disease patients but not in those who suffered from mild cognitive impairment [16].

When aging is not complicated with any detectable neurological disease, the cholinergic neurons of this complex undergo moderate degenerative changes, resulting in cholinergic hypofunction that has been related to the memory deficits progressing with age. Importantly, it has been found that in normal aging nearly no neural cell loss occurs, which is suggestive that functional decline associated with aging is mediated by mechanisms other than those involved in the development of dementia (reviewed in [17]). One of these relates to the fact that levels of ACh in the brain appear to decline with age. A general idea behind the use of choline donors (such as citicoline) as so-called „memory boosters” has been presented, according to which more ACh could be produced if the brain had more of choline, the substrate needed to make ACh [18].

In addition to being the precursor to ACh, choline also serves as the precursor of phospholipids sphingomyelin and phosphatidylcholine (PtdCho), which are structural components of cell membranes. PtdCho is also an important constituent of myelin, which in the brain is produced by oligodendrocytes. Myelin sheaths wrapped around axons enable rapid saltatory conduction of action potentials and contribute to the maintenance of axonal integrity. Human as well as rodent brain myelin is characterized by a high lipid content, its dry mass consisting of 70-85% lipids. Circa 45% of these lipids are phospholipids, mostly PtdCho (see [19] and the references cited). Very recent studies provided evidence that the experience-dependent formation of myelin in the circuits encoding memory is an important aspect of how memories are consolidated and recalled (reviewed in [20]). These observations further support the idea that adequate choline availability plays an important role in memory.

Humans deprived of choline develop liver damage, as clearly shown by „depletion-repletion” studies in patients dependent on parenteral nutrition. Only one pilot study of this type has been reported concerning choline and human memory. Buchman et al. [21] described memory abnormalities (delayed visual recall, verbal learning, and visual scanning ability/ psychomotor speed) in a group of eleven patients who before entry in the study covered more than 80% of their nutritional needs for at least 12 weeks with parenteral nutrition. The patients were randomly assigned to receive their usual total parenteral nutrition regimen that did not contain choline (n = 6) or their usual regimen plus 2 gram of choline chloride (n=5) and subjected to a battery of memory tests at the beginning of the experiment and after 24 weeks of supplementation with citicoline or placebo. Although the study group was very small, choline supplementation compared with placebo resulted in improvement of delayed visual recall, verbal learning, and visual scanning ability/psychomotor speed.

In agreement with the observations mentioned above, significant associations in the adults and elderly between dietary choline intake or plasma choline level and performance in memory and cognitive functions have been reported in 2 large population studies.

In 1,391 non-demented adults (mean age 60.9 years) Poly et al. [22] found the relation between dietary choline intake, brain morphology and cognition. Daily choline intake was estimated using the Harvard Food Frequency Questionnaire (FFQ, a validated method for assessing habitual dietary intake over a specified period of time). The results obtained were correlated with the Wechsler Memory Scale outcome measures of neuropsychological tests assessing different cognitive domains: verbal memory, visual memory, verbal learning, attention and executive function. Multivariate adjusted linear regressions performed to assess the linear trend in individual neuropsychological items across quartiles of choline intake showed that higher choline intake was significantly and positively related to verbal memory in both the immediate and delayed recall (adjusted average change across choline quartiles 0.28, 95% CI:0.05-0.51, P=0.02 and 0.30, 95% CI: 0.13-0.46, P=0.01, respectively). Higher choline intake was also significantly positively related to visual memory in both the immediate and delayed recall (adjusted average change across choline quartiles 0.25, 95% CI:0.05-0.45, P=0.01 and 0.26, 95% CI: 0.05-0.47, P=0.01, respectively).

In the other aspect of the Poly et al. study, the FFQ estimates of daily choline intake were correlated with magnetic resonance imaging (MRI) measures of the brain white matter hyperintensity volume (WMHV). Choline intake was inversely related to log-transformed WMHV (average change in log WMHV per 1-unit change in choline was 20.05, 95% CI:20.10-20.01, P=0.02), and to presence of large WMVH (OR: 0.56; 95% CI: 0.34, 0.92; P=0.01). MRI-detected white matter hyperintensities (WMHs), also known under the term leukoaraiosis, are detected in patients who have impaired performance in a wide range of cognitive domains characterized mainly by detrimental changes in processing speed and executive function [23]. However, leukoaraiosis is a frequent finding also in non-demented elderly people [24,25].

In the study of 2,195 subjects aged 70-74 years [26] it has been found that low plasma free choline concentrations were cross-sectionally associated with poor performance in validated tests for global cognition, sensorimotor speed, perceptual speed, and executive function after adjusting for other factors known to influence cognition. Compared with low choline concentrations, high plasma choline (>8·4μmol/l) was associated with better test scores in the Trail Making Test part A (56·0 v. 61·5, P<0·004), modified versions of the Digit Symbol Test (10·5 v. 9·8, P<0·005) and Mini Mental State Examination (11·5 v. 11·4, P<0·01).

The other recent study [27] was based on the data from the US National Health and Nutrition Examination Survey (NHANES), 2011-2014. The analysis, which included data on 601 female and 530 healthy male seniors (aged >60 years), aimed at finding whether inadequate intake of micro-nutrients, as defined by the Institute of Medicine’s (IOM) Recommended Dietary Allowance (RDA) according to gender, is associated with lower working memory performance. Data on diet and supplement exposure were collected in person for the 24-h (midnight-to-midnight) period prior to administration of the survey, and daily intake of 8 micronutrients, namely vitamins B2, B6, B12, C, choline, folate, niacin, and zinc were assessed. Working memory was assessed with the Wechsler Adult Intelligence Scale (WAIS III)’s Digit Symbol Substitution Test (DSST). Logistic regression was used to estimate unadjusted and adjusted odds ratios for having a memory performance score in the lowest quartile for individuals with inadequate compared to adequate RDA levels. Unlike in the other studies, the correlation between inadequate choline consumption and less efficient memory was gender-dependent. The odds ratio of being in the lowest quartile of DSST scores when individual under-consumed choline was highly statistically significant in males (P<0.0001), but not significant in females.

The aforementioned population studies strongly suggest a cause-and-effect relationship between dietary choline availability and maintenance of memory function in middle-aged and elderly, cognitively healthy (non-demented) adults.

## Uridine as a precursor of neuronal cell membrane phospholipids

In humans, cytidine released from citicoline following its oral intake is in circulating blood plasma readily and almost completely converted to uridine due to plasma cytidine deaminase activity [28,29]. Uridine belongs, along with choline and docosohexanoic acid, to the compounds present in circulating blood which are required for brain phosphatide synthesis [30]. Higher uridine levels are thought to exert a positive effect on synaptic function, support synaptic membrane formation and alleviate synaptic dysfunction (see [31], and the references cited).

Agarwal et al. [32] reported an effect of uridine on membrane phospholipid precursors in the brains of 17 healthy male adults using phosphorous magnetic resonance spectroscopy (31P-MRS). Study participants took 1 g of uridine or placebo two times daily for seven days. Sustained administration of uridine appeared to increase brain phosphomonoesters in healthy subjects.

No study has been published to date showing that uridine *per se* supports memory in healthy elderly persons, but uridine (along with choline) is an ingredient in some proprietary products indicated for the dietary management of failing memory in the early Alzheimer’s disease (eg. Souvenaid® [33]).

## Choline uptake by the human brain and its metabolic disposal following intake of choline compounds

Magnetic resonance spectroscopy (MRS) is a set of non-invasive techniques that allow for the quantification in the brain of several major metabolites. Two types of MRS techniques used in the studies assessing the effects of diet on cognition are proton resonance spectroscopy (1H-MRS) and phosphorous resonance spectroscopy (31P-MRS) [34]).

1H-MRS allows for quantification of the sum of water-soluble choline compounds, namely phosphor-choline (PC) and glycerophosphocholine (GPC), with small or negligible contributions from free choline and acetylcholine. The 1H-MRS signal does not include a contribution from cellular membrane-bound choline-containing phospholipids such as phosphatidylcholine (PtdCho) despite the fact that these comprise approximately 96% of brain choline-containing compounds. However, changes in phosphatidylcholine are reflected by changes in the H-MRS signal because PC is as a precursor of PtdCho, and GPC is a catabolite of PtdCho. 31P-MRS allows for the indirect quantification of membrane phospholipid metabolism. In this regard, the level of phospholipid anabolites (phosphomonoesters, PME), including phospholcholine (PC) and phosphoryl-ethanolamine (PE), is indicative for membrane synthesis, while the level of phospholipid catabolites (phosphor-diesters, PDE), mainly glycerolphosphocholine (GPC) and glycerolphosphoethanol-amine (GPE), is a measure of membrane breakdown. Consequently, the ratio of PME to PDE (PME/PDE) can be used to quantify membrane turnover.

Two 1H-MRS human studies showed the increase of 1H-MRS signals following oral choline bitartrate. Stoll et al. [35] followed the effects of ingesting a single dose of choline bitartrate equal to a free choline dose of 50 mg/kg body weight in four healthy young volunteers of unspecified age. The basal ganglia was chosen as the region to study because of its rich innervation with cholinergic neurons. The authors found that the choline resonance signal (expressed as a ratio to the creatine resonance signal) did not significantly change 1.25 hour after choline intake (mean change 7.7±15.1%), but it significantly increased at 3 hours (mean change 94.2±35.5%, *P*=0.008). In the follow-up investigation Cohen et al. [36] measured plasma and brain choline levels with 1H-MRS after a single dose of choline bitartrate in a dose equal to a free choline dose of 50 mg/kg body weight in young and aged healthy subjects. The results showed that, despite a similar choline increase in plasma in both groups, brain levels of choline measured by 1 H-MRS were lower in the aged group, suggestive of a decrease in choline uptake into the brain with age. Given the role of choline in cholinergic neuronal function supporting memory, the result was interpreted as a sign of reduced uptake of choline that may be involved in age-dependent altered memory function.

However, two other studies did not confirm the aforementioned observations. Tan et al. [37] claimed to repeat as closely, as possible, on a group of young healthy volunteers (average age 26 years) the protocol of the aforementioned study by Cohen et al. but failed to find a change in the choline resonance signal from basal ganglia as well, as from gray and white matter of parietal cortex, at 3h and 5h following oral intake of choline bitartrate. In this study along with 1H-MRS the authors used 31P-MRS to record four principal phosphorylated choline metabolites prior to and 3 h after choline ingestion, but they also found no change. Dechent et al. [38] used 1HMRS to follow choline resonance signals in young volunteers following ingestion of choline bitartrate given as a single dose of 50 mg/kg body weight, or phosphatidylcholine (soy lecithin), 16 g dose given twice daily for four weeks. Neither short-term choline nor monthly phosphatidylcholine supplementation resulted in statistically significant changes of choline resonance acquired from four different locations in the brain, paramedian parietal gray matter, parieto-occipital white matter, central cerebellum and thalamus.

In the last study in which H-MRS was used to follow choline resonance signal from the human brain following choline bitartrate ingestion (dose equivalent to 50 mg choline per kg body weight) [39], the authors for 2.5 h continuously acquired the spectroscopic data from the left putamen of 11 young male subjects. When the data obtained were compared with the effect of placebo ingestion, small increases of choline resonance signals relative to creatines or to N-acetylaspartate signals were noted following choline intake. The authors concluded that their study demonstrated that in humans, as in animals, ingested choline does get into the brain and may be important for the normal synthesis of acetylcholine and phosphatidylcholine.

Compared to the data on changes in brain choline compounds following oral choline or phosphatidylcholine intake which are ambiguous, the MRS data concerning the effects of citicoline ingestion on brain choline compounds are more cohesive. Babb et al. [40] investigated the effects of single doses of citicoline on choline H-MRS signal from the head of caudate and the putamen area in the brain. The study groups comprised six young male volunteers (age 25+3 years) and six older subjects (four males and two females, age 59+3 years). H-MRS spectra were recorded prior to and 3 h after the intake of 0.5, 2 or 4 g citicoline. The main finding of the study was that, in spite of similar increases in plasma choline following citicoline intake in older and younger subjects, the choline resonance signal (expressed as a ratio to the creatine signal) decreased by 4-16% in older subjects (*P*<0.05 after the highest citicoline dose only), whereas in the younger subjects it increased by 12-26% (changes statistically not significant). The authors explained these results as a consequence of older persons encountering decreased brain ability of choline uptake, whereas cytidine uptake was not affected. Increased choline availability from blood following citicoline intake improved brain uptake of choline in older persons, resulting in the increased incorporation of choline together with additional intracellular cytidine into membrane phospholipids not visible by 1H-MRS technique.

In two studies the effects of citicoline intake on brain phosphate compounds were investigated with 31P-MRS technique. Later the same group [41] recorded phosphorus resonance spectra from genu of corpus callosum prior to and after 6 weeks of the oral intake of 0.5 g citicoline daily. Treatment with citicoline resulted in a 7.3% increase of brain phosphodiesters (*P*=0.008), including an 11.6% increase in glycerophosphoethanolamine (*P*=0.002) and a 5.1% (but not significant) increase in glycerophosphocholine. The authors concluded that their study provided the first *in vivo* human data showing increased phosphatidylcholine synthesis in the brain following citicoline administration. Moreover, the correlation between increased concentrations of phosphodiesters and improved verbal learning in healthy older subjects suggested that the administration of oral citicoline may be of use in reversing age-related cognitive changes.

In the second study [42] 31P-MRS technique was employed to observe main phosphorous metabolites in two brain areas, anterior cingulate cortex (ACC) and parieto-occipital cortex (POC) of 16 healthy adults (mean age 47.3 years) following intake of citicoline in a daily dose of 500 mg or 2,000 mg for 6 weeks. Based on the preclinical data the authors hypothesized that citicoline may alter not only resonance-visible phospholipid metabolites, but also resonance signals of phosphometabolites related to energy production and utilization, namely inorganic phosphate, phosphocreatine, and beta-nucleoside triphosphates (β-NTP, which mostly is related to ATP). After 6 weeks of citicoline supplementation the main findings were significantly increased resonance signals of phosphocreatine (*P*<0.02) and β-NTP (*P*<0.05), two phospholipid membrane anabolites phosphocholine (*P*<0.02) and phosphoetanolamine (*P*<0.04), and one phospholipid membrane catabolite glycerophosphocholine (P <0.01). Importantly, citicoline-related changes were detected in ACC, but not in PCC.

Although the MRS data concerning citicoline effects on choline and phosphate metabolites in the brains of adult and elderly healthy humans are fragmentary, they correspond well to the other relevant observations. Significant increase following citicoline supplementation of glycerophosphoethanolamine resonance signal in the genu of the corpus callosum of middle-aged adults, interpreted as a reflection of increased phosphatidylcholine synthesis in this area, corresponds to the observation that citicoline intake attenuated the development of leukoaraiosis in the corpus callosum ([43], see below). The potential importance of signs of improvement in bioenergetics in the anterior cingulate cortex following citicoline intake are underscored with the data showing that during healthy aging ACC is function-wise among the most affected cortical regions [44].

## Intervention trials assessing oral intake of citicoline on memory in middle-aged or elderly persons considered healthy

Four scientific reports have been identified that describe prospective intervention trials designed to assess the effects of oral citicoline on indices of memory (three publications), and on a relevant radiological correlate of memory (leukoaraiosis, one publication).

The first study, which was placebo-controlled [45], was performed with 95 subjects (47 women and 48 men), age 50 to 85 years (mean 67.2±9.3 SD years). Exclusion criteria included, *inter alia*, active medical, neurological, or psychiatric illness, subjects also had to score 26 or greater (out of a possible score of 30) on the Mini-Mental State Examination. There were two stages of the study. The initial stage was randomized, double-blind, placebo-controlled, during which the subjects took either placebo or citicoline, 500 mg twice daily for 3 months. Citicoline-treated subjects had significantly higher mean blood plasma choline concentrations than placebo-treated subjects. Citicoline-treated subjects, but only those with relatively inefficient memories, exhibited a trend for improvement in the standardized measure of memory function that resembled the memory requirements of real life, which consisted of immediate recall and later recall of the passages from the Logical Memory subtest of the Wechsler Memory Scale and the Wechsler Memory Scale Revised. In the second part of the study a subgroup of 27 subjects with relatively inefficient memories received 1,000 mg twice daily or a placebo for 2 months, with a 10-day washout period prior to crossing over, and improvement of verbal memory functioning following citicoline intake was clearly confirmed.

In the second placebo-controlled, crossover study [46] the effects of oral citicoline (daily dose 500mg or 1,000mg) taken over 4 weeks by 24 elderly, non-demented subjects (18 male and 6 female, mean age 66.12 years, mean Mini-Mental Score 31.69) on memory performance were evaluated using neuropsychological tests assessing the following memory tasks: word recall, word recognition, immediate recall and delayed recall of objects, and recognition of objects. Prior to testing the effects of citicoline, the memory performance of the participants was compared to that of 24 younger persons (15 male and 9 females, mean age 29.20 years, mean Mini-Mental Score 34.41), and the memory of the older group was significantly less efficient in all 5 tests (*P*<0.001, except of P<0.005 in word recognition test). Following 4 weeks of citicoline treatment improvement was noted in 2 of 5 memory tasks, namely in word recall (*P*<0.02) and delayed object recall (*P*<0.001).

In the third study [43] Diffusion Tensor Imaging (DTI) technique has been used to investigate the effect of citicoline on the indices of the network connectivity of the corpus callosum in patients with leukoaraiosis. DTI allows to quantitatively describe water molecule diffusion patterns in the brain white matter which are related to microscopic details of tissue architecture, such as the integrity of myelin [47]. After excluding stroke history, central nervous system and mental diseases, neurodegenerative diseases, and addiction to alcohol or drugs, 30 persons were recruited diagnosed with moderate to severe leukoaraiosis. The participants were voluntarily assigned to the citicoline group (n=14) and the control group (n=16). In the citicoline group, patients received a daily dose of 600mg citicoline for one year, whereas the control group received no citicoline. Quantitative DTI of three parts of the corpus callosum, the genu, body, and splenium, were performed on recruitment and after one year of citicoline treatment, and the results were presented as the estimates of mean diffusivity (MD) which measures the overall water diffusivity in the tissue, and fractional anisotropy (FA) which measures the degree of directional restriction of the diffusion of water. Compared to the results obtained at the beginning of the trial, after one-year subjects belonging to the control group displayed increases of MD and decreases of FA in all three locations, indicative of pathology progression. Subjects who received citicoline did display decreases in FA as well, but at all three locations, they were markedly smaller than the changes noted in the control group (*P*=0.005 for genu of the corpus callosum, *P*=0.052 for the body of corpus callosum, and *P*=0.011 for splenium of corpus callosum). Citicoline intake over one year resulted in unchanged MD values, the effect was clearly different from that seen in the control group (*P*=0.007 for genu of the corpus callosum, *P*=0.002 for the body of corpus callosum and *P*=0.002 for splenium of corpus callosum). These data are suggestive of that in subjects with leukoaraiosis, citicoline may attenuate the damage to the axons and myelin and promote the repair of the corpus callosum.

In the fourth, most recent, randomized, double-blind, placebo-controlled study [48], the effects of oral citicoline intake on memory in healthy elderly subjects displaying age-associated memory impairment (AAMI) were evaluated. The study was carried out in compliance with the protocol and in accordance with Good Clinical Practices (GCP). Males and females aged between 50 and 85 years were randomized to receive placebo (n = 51) or citicoline (n = 49; dose 500 mg/day) for 12 weeks. Cognitive performance was assessed at baseline and end of the intervention using computerized tests developed and validated by the MRC and Brain Sciences Unit, University of Cambridge, Great Britain. These tests assess working memory, short-term spatial memory, short-term verbal memory, episodic memory, selective attention, and sustained attention. Compared with those taking placebo, participants taking citicoline demonstrated a significant improvement in episodic memory and overall memory assessed by the composite memory score. The authors concluded that their results were convergent with two previous studies indicating positive effects of citicoline on memory in middle-aged and elderly persons.

## The effects of citicoline on memory in patients suffering from chronic neurological diseases

Before citicoline (inner salt) was accepted as a food constituent, citicoline (both inner salt and sodium salt) had been extensively used as a prescription drug used to treat neurological diseases, both acute (ischemic brain stroke, brain trauma) and chronic (dementias, Parkinson’s disease). In multiple prospective trials, patients suffering from various memory-impairing diseases have been treated with citicoline.

A final version of the Cochrane review concerning citicoline for cognitive and behavioral disturbances associated with chronic cerebral disorders in the elderly [49] included the analysis of 14 randomized, placebo-controlled studies on aged individuals with symptoms ranging from memory disorders to vascular mild cognitive impairment, vascular dementia, or senile dementia. The main findings of this review had been summarized [50] as follows: Duration of studies ranged between 20 and 30 days, 1 study lasted 6 weeks, 4 studies lasted 2 and 3 months, and 1 study lasted 12 months. Multiple doses and different inclusion criteria and outcome measures were used. Overall results (884 patients) showed evidence of the benefit of citicoline on memory and behavior but not on attention. There was a significant improvement in the global impression of change in comparison with the placebo group. Odds ratio (OR) for global improvement following active treatment compared to placebo was 8.89 (95% CI, 5.19-15.22; P<0.001), indicating a rather strong drug effect. Importantly, as noted by Fioravanti and Buckley [51], when results of memory tests were taken into account, the effect of citicoline on memory was also significantly different from the placebo effect, being not specifically dependent on the pathogenesis of the cerebral disorder (effect size 0.19; confidence interval [CI] 95% 0.06, 0.32; p<0.005). Moreover, when only cerebrovascular disorders studies were pooled together (for a total of 675 patients), the homogeneity and entity of results was about the same (effect size 0.22; CI 95% 0.07, 0.37; p<0.004). This indicates that the positive effect of citicoline on memory does not depend on the type of underlying brain pathology, and the mechanism responsible is probably the same as in cognitively unimpaired older adults.

Besides the data reviewed in the aforementioned Cochrane analysis, the results of an Italian open multicenter study (the IDEALE study) were published in 2013 [52]. Its aim was to assess the effectiveness and safety of oral citicoline in elderly people with mild vascular cognitive impairment. Of note is that the authors considered citicoline as a dietary supplement. The study group comprised 349 patients, 265 of whom (122 men and 143 women) were taking citicoline, and the remaining 84 who did not take it served as the control group. Inclusion criteria were age at least 65 years, subjective memory complaints, Mini-Mental State Examination (MMSE) score not less than 21 indicating a mild degree of cognitive impairment, and neuroradiological evidence of brain vascular lesions. Although memory is only one of few domains tested in MMSE, which also includes tests of orientation, attention, language and visual-spatial skills, the study is unique because the treatment has been conducted for a period of 9 months, longer than in most previous trials. Citicoline-treated patients encountered unchanged MMSE scores (22.4±4 at the beginning, 22.7±4 at 3 months and 22.9±4 at 9 months), whereas controls presented deterioration of MMSE score (21.5±6.9 at the beginning; 20.4±6.6 at 3 months and 19.6±6.3 at 9 months). The difference between the groups was statistically not significant at the beginning of the study but reached high significance at both 3 and 9 months of the study, P<0.0001.

## Conclusions

Leading world markets are currently flooded by hundreds of dietary supplements intended for enhancing brain health and improving cognitive performance. Such products are targeted to the aging persons concerned with or experiencing cognitive decline as well as to healthy adults seeking to improve or enhance memory performance or prevent a cognitive decline. Many authors indicate that these “memory boosters” should not be trusted because scientific evidence behind them is inadequate, studies are inconsistent and imprecise, and many trials are methodologically flawed (see for example, [53]).

A good example is the case of polyphenols, a diverse group of substances encountered in plant-based foods and considered micronutrients. Polyphenols (flavonoids and related phenolic compounds) are so-called secondary plant metabolites, i.e., compounds not necessary for the growth and reproduction of plants. After ingestion dietary polyphenols, which usually are sparingly water-soluble, appear in the circulatory system not as the parent compounds but as more water-soluble phase II metabolites, and their presence in plasma after dietary intake rarely exceeds nM concentrations [54]. Their role as components responsible in part for the putative protective effects of vegetable-rich diets against many chronic diseases has become an important area of human nutrition research.

The issue of polyphenols and human memory and cognition has recently been the subject of several systematic reviews and meta-analyses. In one of them [55], it was stated that the evidence from clinical trials is by no means conclusive, although there is tentative support for a relationship between regular polyphenol intake and cognitive benefits. The other [56] provided a bit more optimistic conclusion: of 66 randomized controlled trials testing the effects of polyphenols on memory, 33 found a significant improvement on at least one memory outcome measure, while 30 did not find any significant effects, and three reported a worsening.

**Table 1:** Converging lines of evidence for positive effects of citicoline on memory in cognitively normal middle aged and elderly humans.

Line of evidence	Main findings	References
**Epidemiological/cohort studies**	Reciprocal association between choline availability and memory efficacy	15, 16, 17, 20
**Basic science/animal studies**	Citicoline is the efficacious source of bioavailable choline and cytidine/uridine	5, 6
**Human „proof of concept” studies**	Citicoline supplementation improves brain MRS and MRI biomarkers	34, 35, 36, 37
**Human intervention studies**	Citicoline supplementation positively influences human memory	39, 40, 42, 43, 44, 45, 46

How can we compare the strength of scientific evidence for the positive effects of citicoline and polyphenols on human memory? A scholarly article published recently by a group by Kirk Daffner from the Department of Neurology, Brigham and Women's Hospital, Harvard Medical School [57] prompted us a promising methodology to perform such a comparison. The said article concerns the issue of promoting successful human cognitive aging. The authors discuss the advantages and limitations of four different lines of evidence used to evaluate whether a proposed factor or intervention really may have an impact on cognitive aging. These are: 1) epidemiological/cohort studies, 2) animal/basic science studies, 3) human proof-of-concept studies, and 4) human intervention studies. To prove that a factor or intervention under consideration is efficacious, findings along all these lines shall converge. As we were trying to justify, in the case of citicoline and human memory, these four lines of evidence nicely converge (see Table 1). In the case of polyphenols and human memory, such convergence seems to be much less clear.

Currently, the mainstream treatment of dementing diseases is based on the use of acetylcholinesterase inhibitors (donepezil, rivastigmine, and galantamine), and/or a low affinity NMDA antagonist memantine. The use of these prescription drugs is burdened with side effects, and it is unlikely that they will be prescribed to cognitively competent persons suffering only from mild age-related memory troubles. However, it is worth mentioning a few clinical studies (reviewed recently by Gareri et al. [58]) which indicated that treatment of patients suffering from early Alzheimer’s disease or mixed dementia with citicoline combined with memantine and/or an AChE inhibitor resulted in slight but statistically significant improvement, whereas treatment with memantine and/or an AChEI, drugs formally indicated for the early dementias, resulted is a slight but statistically significant worsening of MMSE scores at 3 or 9 months. Although these studies were neither prospective, nor placebo-controlled, differences between avereage MMSE scores between the groups were impressive. For example, in the CITIMEM study [59] supplementing metantine with citicoline for 9 months resulted in the improvement of the average MMSE score from 16.2 to 17.7, whereas treatment with memantine alone resulted in the average MMSE score deterioration from 16.6 to 14.6. Such data are indicative of citicoline effect being larger than the suggested threshold of a minimal clinically important difference in an Alzheimer’s disease clinical trial [60]. These observations provide further confirmation that citicoline shall be seriously considered as an agent providing support for failing memory in middle-aged and elderly persons.
